# The Covid-19 pandemic seen from the frontline

**DOI:** 10.1590/S1677-5538.IBJU.2020.S123

**Published:** 2020-07-27

**Authors:** Luz Elena Ojeda Carmona, Maria Del Carmen Córdoba Nielfa, Alvaro Luis Diaz Alvarado

**Affiliations:** 1 Cantabrian Health Service Cantabria Spain Primary Care Emergency Service, Cantabrian Health Service, Cantabria, Spain; 2 Albacete University Hospital Complex Castilla-La Mancha Spain Albacete University Hospital Complex, Castilla-La Mancha, Spain; 3 Sierrallana Hospital Cantabria Spain Emergency Service, Sierrallana Hospital, Cantabria, Spain

**Keywords:** spike protein, SARS-CoV-2 [Supplementary Concept], Primary Health Care, Emergencies

## Abstract

COVID-19 disease caused by infection with the SARS-CoV-2 virus produces respiratory symptoms, predominantly of the upper airways, which can progress to pneumonia after 7 days with persistent fever, cough and dyspnea, and even develop a syndrome of acute respiratory distress (ARDS), multi-organ failure and death. Since COVID-19 disease was declared by the WHO there has been a redistribution of the healthcare system for these types of patients, especially in the front line, which is, in primary care, emergencies and in intensive care units (ICU). In primary care, the fundamental role is the diagnosis of the suspected patients, follow-up mainly by telemedicine (specially telephone calls) to detect warning signs in case of worsening and subsequent referral to the emergency department; as well as explaining home isolation measures. In the emergency department, it is included the management of suspicious cases and, if it any risk factor is found, complementary tests are carried out for precise diagnosis and admission assessment; In case of oxygen saturation <95% and poor general condition, valuation is requested for admission to the ICU. Depending on the severity of the patient, he/she would be or not a candidate for invasive mechanical ventilation, which must be performed by trained personnel to prevent the spread of the infection minimizing the risk of contagion. ARDS's treatment strategies include pulmonary protection ventilation, prone position, recruitment maneuvers and, less frequently, oxygenation by extracorporeal membrane. Among the specific treatments for COVID-19 stand out mainly drugs to reduce viral load, although sometimes specific drugs will be needed to treat hyperinflammation, hypercoagulability and concomitant infections.

One of the goals to be achieved is for patients to recover and be able to successfully return to work; for this purpose, an adequate physical and psychological rehabilitation program is essential, as about 50% have symptoms of anxiety and depression.

## INTRODUCTION

The novel coronavirus disease 2019 (COVID-19) is caused by the severe acute respiratory syndrome coronavirus 2 (SARS-CoV-2) and is currently a pandemic declared by the WHO on March 11, 2020, therefore, a public health emergency (1). As the pandemic has intensified, health systems have established protection standards and technical follow-up documents for the management of patients in different areas, including primary care, hospital emergencies, in the front line of medical care and transfer to the intensive care unit (ICU) of patients with serious complications; with emphasis on measures to protect patients and health personnel against the virus. In many cases, the desire for widespread masking is a thoughtful reaction to pandemic anxiety (2). Based on recent information, we provide a summary regarding to the diagnosis, management and treatment of the COVID-19 patient from the first line: Primary health care emergency and intensive care unit.

The following is a brief summary of the virus causing the disease(l, 3, 4):

1.1. It is a zoonotic virus, SARS-CoV-2 with the first case in Spain in late January 2020.
1.2. Binds to angiotensin-converting enzyme 2 receptors in alveoli.
1.3. Sensitive to heat and disinfectants.
1.4. Transmission by respiratory drops and contaminated fomites.
1.5. No demonstrated airborne or feces transmission.
1.6. On plastic surfaces it lasts 72 hours, stainless steel 48 hours, cardboard 24 hours, copper 4 hours, in air after aerosols 2 hours.
1.7. Prodromal phase 1-2 days before symptoms start, up to 14 days if moderate or severe.
1.8. Transmission of 1.5 - 4 cases by an infected.
1.9. COVID-19, the serial interval is estimated at 4-4-7-5 days, which is more similar to severe acute respiratory syndrome (SARS).
1.10. Estimates suggest that about 80% of people with COVID-19 have mild or asymptomatic disease, 14% have severe disease and 6% are critically ill.

## EVIDENCE ACQUISITION

For carrying out the systematic search of the literature, we used online databases such as PubMed, Cochrane, Google Scholar, UpToDate; Among the search criteria referring to COVID-19, we highlight RCTs published in the last 3 months, in humans, in English and Chinese languages; In the search criteria regarding supportive treatment as pharmacological, the search was extended to the last 15 years. The most frequently used descriptors were “coronavirus infections”, “Primary Health Care”, “Emergency Medical Services”, “Intensive care unit”, “pneumonia”, “Respiratory Distress Syndrome Adult”. As well as the publications of the Technical Documents of the Ministry of Health of the Government of Spain on the diagnosis, management and treatment of COVID-19.

## 1 - CLINICAL MANIFESTATIONS

The WHO mission report in China describes the most common symptoms and signs of laboratory-confirmed cases, including fever (87.9%), dry cough (67.7%), asthenia (38.1%), expectoration (33.4%), dyspnea (18.6%) (5).

In Spain the most common symptoms reported are fever, cough, dyspnea and chills; 40% developed a digestive clinic (diarrhea or vomiting). Men have a higher prevalence of fever and dyspnea, while sore throat and digestive clinic are significantly more common in women.

It is characterized by(5):

1.1. COVID-19 syndrome: Coronavirus disease 2019 (COVID-19) is a potentially serious acute respiratory infection caused by severe acute respiratory syndrome coronavirus 2 (SARS-CoV-2). The set of clinical manifestations caused by SARS -CoV-2 infection includes from mild respiratory symptoms to severe pneumonia with respiratory distress syndrome, septic shock and / or multi-organ failure. The development of these complications largely depends on the patient's prior immune status.
1.2. They are classified into:
  1.2.1. Asymptomatic Infection: state in which there are no symptoms and the transmissibility of SARS- -CoV-2 can occur in this condition up to a minimum of 2 days before the onset of symptoms.
  1.2.2. Mild cases: Characterized by an upper respiratory infection (fever, dry cough, odynophagia, nasal congestion, headache, myalgia). Symptoms are generally present for about two weeks until recovery (20 days).
  1.2.3. Moderate cases: characterized by respiratory symptoms and pneumonia, pulmonary infiltrates that progress in 24 to 48 hours, these patients may or may not be admitted.
  1.2.4. Severe cases: those that require hospitalization, who present dyspnea, tachypnea, 02 Saturation <93%; of which approximately a third require admission to the ICU (Adult Respiratory Distress Syndrome, Sepsis, Shock). Most of these complications appear 7 days after the onset of symptoms and recovery in 3 to 6 weeks.

## 2 - DIAGNOSIS

2.1. The microbiological diagnosis of COVID-19 is carried out using the Polymerase Chain Reaction (PCR) technique, determining the Ribonucleic Acid (RNA) of the SARS-CoV-2 virus, therefore, it is the diagnostic test per excellence.
2.2. Rapid antigen or antibody detection techniques are not considered adequate for diagnosing acute infection. ELISA-type serology or other high-performance immunoassay techniques are not indicated by themselves for diagnosis in the acute phase of the disease (6). There is the total antibody test indicated in symptomatic patients with several days of evolution and the test that differentiates immunoglobulin M (IgM) and immunoglobulin G (IgG) indicated in both symptomatic and asymptomatic patients to know their immunological situation (healthcare professionals, nursing homes, contacts).
2.3. The sample studied is blood obtained from venous blood or a lancet digital puncture (4).
2.4. These diagnostic tests will be carried out on symptomatic patients in the hospital setting with moderate or severe involvement, whether or not they have negative PCR: If the result is positive, the diagnosis is confirmed (recent or past infection) and if the result is negative, PCR will be performed (2).
2.5. In the non-hospital setting, mild symptomatic cases will be made with priority to nursing homes.
2.6. In the community setting, it may be possible to carry out a rapid diagnostic test in patients with high clinical suspicion and several days of symptom evolution.
2.7. Rapid tests in healthcare professionals: It will be carried out on those professionals with clinical suspicion of COVID-19 with negative PCR and more than 7 days after the onset of symptoms in order to confirm the diagnosis (Table-1).
2.8. Professionals without COVID-19 symptoms: Rapid test must be carried out with the purpose of early detection of asymptomatic cases that might be transmitters of SARS- CoV-2 (Table-2).

**Table 1:** Usage of rapid tests in symptomatic professionals. April 2020.

Outcome	Interpretation	Action Guideline
IgM + IgG +	COVID-19 confirmed	Repeat PCR
IgM + IgG -		
IgM - IgG +	Contact with COVID-19	Assess repeat PCR based on symptoms
IgM - IgG -	Discharged for COVID-19 (if after more than 14 days).	If the 14 days have not passed and there is suspicion clinical, repeat PCR and rapid test.

**Table 2:** Rapid tests in asymptomatic professionals. April 2020.

Outcome	Interpretation	Action Guideline
IgM - IgG -	Discharged for COVID-19	
IgM - IgG +		
IgM + IgG -	COVID-19 cannot be ruled out	PCR will be performed
IgM + IgG +		

## 3 - DETECTION AND MONITORING OF THE PATIENT WITH SARS-CoV-2 IN PRIMARY CARE

3.1 Identify suspected cases, diagnose, treat and isolate them (mild cases), explaining the isolation measures at home (ventilated site and preferably use of a single bathroom) (7) and provide continuous care giving confidence and security to the patient. No proof of detection of SARS -CoV-2 in these patients. 80% of patients infected will be mild and will not require admission.
3.2 In the monitoring of mild cases, the appearance of warning signs, especially from 7 - 8 days of evolution such as worsening cough, shortness of breath and fever more than 7 days; and if any appears, patients should be transferred to the hospital for evaluation.
3.3. In primary care, treatment is symptomatic; do not give empirical antibiotics unless there is a clinical suspicion of overinfection. In those cases azithromycin or levofloxacin is recommended.
3.4. Regarding the removal of isolation in minor cases, it is carried out at 7 days without clinical symptoms and/or 20 days since the opening of the follow-up episode.
3.5. Confirmed patients who have required hospital admission and who have been discharged with positive PCR must maintain home isolation and monitor their clinical situation for at least 14 days from hospital discharge and perform confirmatory negative PCR; If it turns out positive, isolation is carried out for 14 more days and at least 7 more days for a new PCR (7).
3.6. The population is informed of the importance of avoiding to go out from their home, just for essential activities, hygiene measures and social distancing.

## 4 - MANAGEMENT OF THE PATIENT WITH SARS- -COV-2 IN EMERGENCIES:

**Figure 1:**
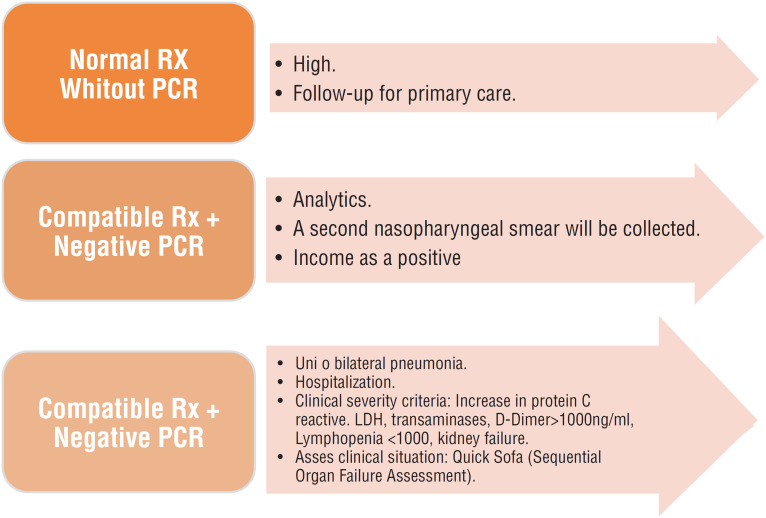
Situations of chest X-ray and its performance in the emergency department. April 2020.

4.1. General population:
  4.1.1. In cases with mild symptoms (do not require hospital admission) home isolation and care follow-up were indicated previously (8).
  4.1.2. General guidelines for the evaluation of patients with infection acute respiratory in the Emergency department (Table-3).
  4.1.3. In all patients with respiratory infection when is necessary hospital admission, PCR for COVID-19 will be requested, and must remain in isolation (Table-4).
  4.1.4. Emergency Treatment: Therapeutic measures will be used according to the clinical severity of the patient (Table-5).
  4.1.5 In summary, it is concluded that all suspicious patients (fever, cough, dyspnea) who go to the emergency room, ventilatory mask and isolation, anamnesis and vital signs (02 saturation> 95%) should be the standard of care; if the patient is > 60 years old and do not present any risk factors (> 65 years, high blood pressure [HEP], Diabetes Mellitus [DM], Obesity, Heart disease, Disease Pulmonary, Renal, Liver, Immunosuppression) he/she is discharged with follow-up for primary care and home treatment. If the patient have any clinical alteration, chest Rx is requested, if it is normal but with O2 saturation <95%, PCR is performed with blood analysis, Legionella antigen and Pneumococcus antigen in urine, to the awaiting result will be in the emergency room. In cases where the chest Rx is altered the patient is entered or discharged according to clinical symptoms, if not presents risk factors or Fine (Pneumonia Severity Index [PSI]) I-II it is suggested to individualize (Figure-1).
    In cases that present a Fine III or risk patient, request ICU assessment.
  4.1.6. Regarding thromboprophylaxis, patients with COVID-19 without pneumonia receive home treatment (Table-6) (9).
4.2 - Children:
  The COVID-19 symptoms in children are generally mild characterized by predominantly respiratory symptoms with cough, fever, and / or nasal congestion, as well as gastrointestinal symptoms. They have the same probability of contagion as adults, are a susceptible population and with high difficulty in controlling respiratory infections (disability for personal hygiene measures in minors) and ease for contagion. As well as the presence of asymptomatic infections or very slight that go unnoticed. Pediatric cases are, to date, scarce (10).
4.3. Pregnant:
  4.3.1. Pregnant women are not more susceptible than the general population for the spread of the disease, nor for the development of complications in women without comorbidities (11).
  4.3.2. Complications increase in patients with Human Immunodeficiency Virus (HIV), HBP, infection, lung diseases, receiving corticosteroid treatment or immunosuppressants. There is few scientific evidence for COVID-19 effects during the first and second trimester of pregnancy.
  4.3.3. There is no clear evidence of intrauterine transmission, and there is a risk of developing premature deliveries, loss of fetal well-being, and delayed intrauterine growth although they are infrequent.
  4.3.4. The presence of SARS -CoV-2 in breast milk has not been demonstrated (11).
4.4. patient over 60 years old:
  4.4.1. The factors that make this age group at high risk are the comorbidities (HBP, DM, chronic lung diseases, cancer or immunosuppression from another cause), higher concentration of angiotensin converting enzyme 2 (ACE2) receptors, living in a nursing home, along with the changes that take place in the immune system due to aging (12).
  4.4.2. Nursing homes are the most vulnerable places therefore the isolation of the elderly and the control measures are essential in preventing the spread of COVID-19; and therein lies the importance of performing rapid antibody detection tests, since the cornerstone is to perform early identification and it is not necessary to wait for the presence of suggestive clinic.

**Table 3:** Ministry of Health, Consumption and Social Welfare (MSCBS). General measures for evaluation in the emergency department of the general population. April 2020 (8).

Patient <60 years, without fever or respiratory insufficiency (02 saturation and respiratory rate in normal ranges for age, ≥ 96% and <20 breaths per minute respectively) or co-morbidity	Usual evaluation. Registration according to usual criteria. Chest radiography according to the criteria of the clinician. Do not request the PCR for COVID-19 in patients who are going to be discharged.
Patient <60 years, with fever and without respiratory failure (saturation ≥ 96% and respiratory rate <20 breaths per minute respectively) or comorbidity: X-ray will be performed in function of the clinician's judgment	If the patient does not have pneumonia, regular evaluation and discharge according to usual clinical criteria. PCR should not be requested to COVID-19 in patients who are to be discharged. If the patient has pneumonia (regardless of the characteristics of the radiological infiltrate), perform analysis (blood count, coagulation, with D-Dimer and basic biochemistry with protein C reactive, Lactate dehydrogenase [LDH] and transaminases).
Patient > 60 years or with comorbidity	X-ray and analytical (basal arterial blood gas, blood count, D-dimer coagulation, and basic biochemistry with C reactive protein, LDH and transaminases).

**Table 4:** MSCBS. Discharge criteria in patients with Pneumonia due to COVID-19. April 2020 (8).

Pneumonia may be discharged in patients <60 years, with Pneumonia Severity Index (PSI) I-II, without radiological complications or analytical complications, if they do not present immunosuppression or significant comorbidity (including hypertension and diabetes).	Unilobar alveolar pneumonia. Without dyspnea With O_2_ saturation and respiratory rate normal Lymphocyte number > 1200 Normal transaminases Normal LDH D-dimer <1.000

**Table 5:** MSCBS. Emergency treatment in patients with COVID-19. April 2020 (8).

Bronchodilators	Use of pressurized cartridge associated with a spacer chamber (inhalers dried). If there is a need for an aerosol, it must be in a room with negative pressure.
Oxygen therapy	In respiratory failure or shock, oxygen with a filter mask exhaled until a saturation appropriate to the age and state of the patient.
Corticosteroids	Do not administer systemically.
Fluid therapy	Conservative management in severe acute respiratory failure without shock.
Septic shock	Administer antibiotics early.

**Table 6:** Spanish Society of Thrombosis and Hemostasis (SETH). Thromboprophylaxis in patients with COVID-19 who do not require admission. April 2020 (9).

Indication	Medical history of Venous Thromboembolic Disease.
	Thrombophilias.
	Cancer.
	Recent surgery.
	Pregnancy / puerperium.
	Hormone Therapy (withdraw anovulatory contraceptives).
Dose	Adjust according to weight and glomerular filtration rate.
	Enoxaparin: <80 kg 40 mg / dL > 80 kg 60 mg / dL.
	Bemiparin: 50 IU / Kg.
Duration	1-2 weeks. Encourage ambulation within the home.

## 5 - TREATMENT OF SARS-COV-2 PATIENTS ADMITTED TO THE INTENSIVE CARE UNIT

In the different studies published, there is discrepancy in the series regarding the number of patients treated in ICU: from 7.7% (13) to 19.1% (14).

A comprehensive patient assessment is essential to evaluate the possibility of admission in the ICU. Once is decided, the patient will be moved to an isolated cubicle with closed suction system. In addition, it is essential to know the procedures that release aerosols, since extreme cautions must be exercised to avoid contagion. Some of these procedures are described below: nebulization, non-invasive ventilation, manual ventilation with mask and self-inflatable bag, tracheal intubation or tracheostomy, aspiration of secretions, bronchoscopy, cardiopulmonary resuscitation, supine-to-prone position change, daily personal hygiene and cleaning of bowel movements.

As it is known, the virus affects mostly the lung, as it has specific affinity for ACE2 receptors which mostly manifest in type 2 pneumocytes (15). From the first symptom to the onset of dyspnea, it takes about 5 days, and until the onset of Acute Respiratory Distress Syndrome (ARDS) about 10 days.

ARDS is a pathology that arises secondarily to a pulmonary or extrapulmonary injury, that occurs with patchy bilateral infiltrates which are not only due to cardiac pathology. It is characterized by loss of pulmonary elasticity (manifested by decreased compliance values) and ventilation/perfusion mismatch with heterogeneous involvement, coinciding collapsed areas with other hyperinflated ones. This was called “baby lung” (16), referring to the functioning of only a small fraction of the lung in the ARDS.

ARDS of patients with pneumonia by SARS-Cov-2 has some particularities, in fact, some recent reviews consider that the virus may occur in the form of two different ARDS phenotypes (17): the initial one, in which the lung is more compliant and there is less shunt, and another phenotype with a more fibrotic lung and with an important shunt (defined as PaO2/FiO2 <150 ratio), but also with a high dead space. In addition, it is characterized by a vascular disorder with a microthrombotic component, and another one which is secondary to the inflammatory response. Finally, it is important to highlight a problem of hypersecretion, with thick secretions that make it difficult to ventilate these patients.

**Figure 2:**
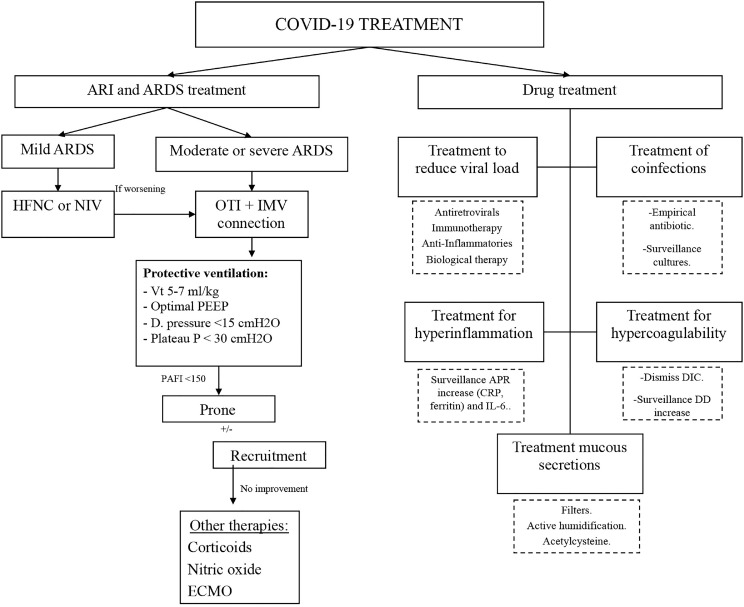
Treatment of SARS-Cov-2.

Therefore, we believe that there would be different targets on which to focus treatment on patients with SARS-CoV-2 who need ICU admission; on the one hand, the treatment of respiratory failure and ARDS, and on the other hand, drug treatment (Figure-2). Details are described below.

5.1. Treatment of respiratory failure and ARDS
  Depending on the clinical situation, we will assess the benefit of initiating non-invasive therapy, such as high-flow nasal cannula (HFNC) or non-invasive mechanical ventilation (NIV); these two procedures were initially discouraged due to their greater susceptibility to generate aerosols, but aerosolization is minimized by avoiding disconnections.
  Another important issue is not to delay intubation if these therapies are not effective. In such cases, a “fast sequence” intubation will be necessary as follows:
  5.1.1. Pre-oxygen with high FiO2. Avoid manual ventilation with mask and Ambu^®^.
  5.1.2. Anesthetic induction with fast-acting drugs in appropriate doses.
  5.1.3. The procedure will be carried out by skilled personnel. The use of disposable devices has to be valuated; a difficult airway should be foreseen.
  5.1.4. Tubes with subglottic suction and suction with closed system shall be used.

As a treatment of the ARDS, the following points stand out:

- Protective ventilation. It consists of ventilating the patient avoiding ventilator-induced lung injury or VILI. To do this, low tidal volumes (Vt) must be ordered: Vt 5-7 mL/kg ideal. In addition, an optimal Positive end-expiratory pressure (PEEP) will have to be prescribed to the patient and his/her pathology. To do this, there are different strategies, but the most suitable is the one performed after pulmonary recruitment, looking for that PEEP that matches the best pulmonary compliance, taking as an optimal PEEP the sum of 2 points (2 cmH20) plus that value that reflects the best compliance. Another protective ventilation strategy is to limit plateau pressures to values below 27 cmH20 and to achieve drive pressure values (DP-Plateau Pressure - PEEP) lower than 15 cmH20, as this last value estimates the transpulmonary pressure, and it is known that its decrease is related to better prognosis (18).
- Alveolar recruitment (AR). It is defined as the re-expansion of previously collapsed lung areas by means of a brief and controlled increase in transpulmonary pressure. The idea of AR is creating and maintaining a collapse-free situation in order to increase the end-expiration volume and improve gas exchange (19). There are different strategies to follow, but the most used, describing it in a very simplified way, is the one based on PEEP increases of 5 to 5 cmH20 maintaining a fixed pressure drive with subsequent descent of the PEEP (to make an optimal PEEP calculation) looking for the best dynamic compliance (19).
- Prone decubitus. It consists of putting the patient face down in a controlled and safe way. With this maneuver, transpulmonary pressures are homogenized and the dorsal areas, which are usually more collapsed and anatomically are the best infused, at being in prone position become more ventilated and recruited, improving the ventilation/perfusion (V/Q) ratio. In addition, it improves diaphragmatic mobility, decreases the weight of the heart on the lungs helping pulmonary expansion and improves hemodynamic, reducing the overload of the right ventricle (20, 21).
- Extracorporeal membrane oxygenation therapy (ECMO). ECMO is a circulatory and respiratory mechanical assistance system which provides cardiac support for a short period of time as a bridge, in the case of respiratory support, until lung recovery or lung transplantation. It is a quick-start assistance through peripheral cannulation and it needs systemic anticoagulation. During ECMO therapy the respirator will be programmed in ultraprotective ventilation mode, to enhance the resting of the diseased lungs. It is essential that ECMO is established in experienced reference hospitals. The role of ECMO therapy in patients with COVID-19 is currently unclear (22).

5.2. Pharmacological Treatment
  - **Antiviral treatment.** It is thought that is paramount to lower the viral load, as it is related to greater inflammation, severity of the disease and higher mortality (23, 24). An indirect marker of the amount of viral load is the persistence of lymphopenia (24).
  - **Treatment of the state of hyperin-flamation**. The reason for the use of these drugs is due to the belief that the underlying pathophysiology of significant organic damage in the lungs and other organs, is caused by an amplified immune response and the release of cytokines, also known as “cytokine storm” (15) or antibody-mediated immunopathology (25).
  - **Surveillance and treatment of coinfections**. Most COVID-19 patients in China received broad-spectrum empirical antibiotics, because it is difficult to distinguish this disease from other bacterial and viral pneumonias, and laboratory diagnosis of COVID-19 takes time (15). It is important to perform cultures and de-escalate when results are available. In addition, it is essential to rule out yeast infections such as Aspergillus, other viruses such as Cytomegalovirus, and bacteria such as *mycobacterium tuberculosis,* since both SARS-CoV-2 itself and the treatments administered can promote such infections.
  - **Surveillance of hypercoagulability state**. There is evidence that anticoagulant treatment improves prognosis in patients with high DD (26). Given that this treatment is not without risk, it is essential to rule out daily the occurrence of disseminated intravascular coagulation and, in patients with DD >6 times normal value, to assess anticoagulation if there is no contraindication.
  - **Treatment of mucous hypersecretion.** In patients who require orotracheal intubation, two high-efficiency filters (in the inspiratory and expiratory branches) will be placed as it allows the exchange of heat and humidity. In addition, treatment with intravenous acetylcysteine will be initiated in any patient who develops broncorrhea.
  - In Table-7, extracted from the Phua J et al. (15) some of the drugs most commonly used as anti-SARS-Cov-2 treatment are included.

**Table 7:** Drugs most commonly used as anti-SARS-CoV-2.

Drug and Dosage	Target	References	Side effects
**REMDESIVIR** – 200 mg once daily in day 1. – 100mg once daily from day 2-10.	NUCLEOTIDE ANALOGUE Inhibits viral RNA-dependent RNA polymerase (RdRp)(15).	– Deemed to be the most promising candidate drug by experts convened in January, 2020, by WHO. – Effectively inhibited SARS-CoV-2, MERS-CoV, and SARS-CoV in vitro (15).	– Possibility of hypotension.
**LOPINAVIR/RITONAVIR** – 400 mg/100mg twice daily for up to 14 days. The timing of administration is during the early peak viral replication phase (initial 7-10 days).	PROTEASE INHIBITOR – It demonstrated in vitro activity against other novel coronaviruses via inhibition of 3-chymotrypsin-like protease (27).	– Second candidate identified for rapid implementation in clinical trials. – A relevant study shows that it is associated with reduced viral load and mortality in an observational study of SARS-CoV (15).	– Prolonged QT interval. – Gastrointestinal side-effects. – It is a CYP3A4 inhibitor.
**HYDROXYCHLOROQUINE** – 400mg twice daily in day 1. – 200mg twice daily from day 2. + **AZITHROMYCIN** – 500mg once daily.	ANTIMALARIAL – block viral entry into cells by inhibiting glycosylation of host receptors, proteolytic processing, and endosomal acidification. – Immunomodulatory effects through attenuation of cytokine production and inhibition of autophagy and lysosomal activity in host cells (27).	– Reduced SARS-CoV-2 load in the nasopharynx of patients with COVID-19, especially when combined with azithromycin (28). Despite these promising results, this study had several major limitations.	– Prolonged QT interval. – Seizures, – Hypoglycemia, – Neuropsychiatric effects, – Retinopathy.
**INTRAVENOUS IMMUNOGLOBULIN** – 400 mg/kg once daily for 3-5 days. In the first 7 to 10 days of infection, when viremia is at its peak and the primary immune response has not yet occurred (27).	IMMUNOTHERAPY	A study of human polyclonal immunoglobulin G (SAB-300) in a mouse model of MERS-CoV found reduced viral lung titers near or below the detection limit in mice infected with MERS-CoV (15).	– In IgA deficiency, risk of anaphylaxis due to anti-IgA antibodies.
**CONVALESCENT PLASMA** In the first 7 to 10 days of infection, when viremia is at its peak and the primary immune response has not yet occurred.	IMMUNOTHERAPY	In SARS-COV-2 is associated with reduction in viral load and improvement in fever, oxygenation, and chest imaging in a case series, but study limited by small sample size, multiple possible confounders, and absence of controls (15).	– Studies of SARS-CoV have not reported serious adverse events (15).
**CORTICOSTEROID** – Metilprednisolone 1mg/kg/dia for 5-7 days, then progressive decrease in dose.	ANTI-INFLAMMATORY Decrease the host inflammatory responses in the lungs.	There is a wide divergence of opinion in the literature on whether corticosteroids should be used in patients with COVID-19, but there is no justification to deny the use of CST in severe life-threatening “cytokine storm” associated with COVID-19(29)	– Adverse effects, including delayed viral clearance and increased risk of secondary infection.
**TOCILIZUMAB** – 8 mg/kg or 400mg iv 1-2 doses.	monoclonal antibody against interleukin-6	Licensed for cytokine release syndrome; hypothetically work against cytokine storm with raised ferritin and interleukin-6 levels due to SARS-CoV-2 (15).	– Increase in upper respiratory tract infections (including tuberculosis) and other infections, hypertension, infusion related reactions, hematologic effects, hepatotoxicity, gastrointestinal perforations.

## 6 - MORBIDITY AND LETALITY RATES

The patients at high risk to developing pneumonia are males, over 60 years, and those with a history of cardiovascular disease and DM.

Early estimates of the lethality rate in Chinese series were around 2%. In Spain the Ministry of Health updates daily the epidemiological summary of confirmed cases of COVID-19 disease reported in Spain and other countries (30).

According to the information available so far, the lethality of COVID-19 in cases reported to REANVE - Red Nacional de Vigilancia Epidemiológica (National Epidemiological Surveillance Network) through the SiViES platform (Surveillance System in Spain) is 7.6% with a range from O% for those under 15 years to 24.3% for those over age 80 years. A specific analysis of deaths shows that deceased patients, vs recovered patients, are significantly older (average age 82 vs 58 years), men are more represented, most often have underlying diseases, pneumonia and other respiratory complications, and more frequently have been hospitalized and admitted to ICU.

If we focus on critical patients, the Chinese series have a great variability, although in the subgroup of patients with ICU admission of the Guan WJ et al. (14) series, 2.3% required mechanical ventilation and 1.4% died.

## 7 - RETURNING TO WORK AFTER THE COVID-19 PANDEMIC

Recovering work activity is synonymous with quality of life, health and return to “normality”; on the contrary, its absence means one more duel to develop (31).

The WHO describes mental health as: “… a state of well-being in which an individual realizes his or her own abilities, can cope with the normal stresses of life, can work productively and is able to make a contribution to his or her community” (32).

After ICU admission, some patients develop Post-Intensive Care Syndrome. In addition to possible physical consequences as a result of the illness as well as the time spent in hospital, bedridden and drugs received, psychological consequences are also common during the first post-ICU year. The incidence of anxiety-related symptomatology is known to be 24%, depression is 28% and post-traumatic stress disorder (PTSD) may account for 22% (33). It is known that 64% of these patients return to work in the two years following the discharge and the quality of life perceived in them is much higher than those who remain unemployed (34).

All of this, in addition to the pandemic situation, makes it necessary to build a psychological support network for all those patients who have required hospital admission for COVID-19 disease and, moreover, for those who have required admission to ICU. Currently, some hospitals have implemented physical rehabilitation and early psychological support programs, initiating them during their stay in the critical care units (35).

## CONCLUSIONS

The COVID-19 disease is currently a pandemic, therefore of worldwide interest for the entire population and posing a challenge for the health professionals in charge, especially those who are on the front line of health. As it is a new virus, there are many unknowns that occur both in diagnosis, management, treatment and subsequent follow-up of these patients. COVID-19 presents with symptoms of cough, fever, shortness of breath, nasal congestion, gastrointestinal symptoms, among others, which can progress to pneumonia and, in some cases, develop an adult respiratory distress syndrome, requiring admission to the intensive care. From primary care, the primary objective is to establish a diagnostic suspicion, generally to follow up by telephone within 24 to 48 hours from the time of the consultation and with surveillance 7 days after the evolution of symptoms, which is when complications occur in the majority of these patients. In hospital emergencies, when a patient suspected of COVID-19 arrives, depending on the clinical state (02 saturation > or <95% and whether or not risk factors), the patient shall be subsidiary to the performance of additional tests for that diagnosis and therefore if is income or not; those hospitalized patients who present acute respiratory failure and bilateral pneumonia will require an ICU admission evaluation, and if it is assumed by the ICU, the different protocols will be applied according to the severity of the clinical picture.
